# Telephone-based support for physical activity: Results and lessons learned during the COVID-19 pandemic

**DOI:** 10.1371/journal.pone.0268429

**Published:** 2022-05-18

**Authors:** Kelly R. Ylitalo, Wendy Cox, Raejone Lucas, Jordan Smith, Kelley Pettee Gabriel, Matthew Rafalski, John Gill, Brock Niceler

**Affiliations:** 1 Department of Public Health, Baylor University, Waco, Texas, United States of America; 2 Waco Family Medicine, Waco, Texas, United States of America; 3 Department of Epidemiology, The University of Alabama at Birmingham, Birmingham, Alabama, United States of America; King’s College London, UNITED KINGDOM

## Abstract

Physical activity is essential to maintain physical and mental well-being. During the COVID-19 pandemic, in-person physical activity opportunities were limited. This paper describes a telephone-based physical activity support strategy among racially/ethnically diverse patients during the COVID-19 pandemic. Adult patients at a large, Federally Qualified Health Center with an on-site exercise facility referral were eligible to transition to telephone support with personal fitness advisors during the pandemic stay-at-home orders. Baseline surveys assessed physical activity and environmental characteristics; follow-up phone calls used motivational interviewing and physical activity goal setting strategies. From March 23-July 23, 2020, 72 patients participated in 270 phone calls, or 3.8 (±2.1) calls per participant. Participants were, on average, aged 51.3 (±11.6) years, 87.5% female, 31.9% Hispanic/Latino, and 47.2% non-Hispanic Black. Patients meeting physical activity guidelines pre-pandemic reported more planned exercise (100.0% vs. 55.3%; p<0.001), exercise days at home (5.0 vs. 1.7; p<0.001), and accomplishment of personal physical activity goals (57.0% vs. 39.7%; p = 0.11) than patients not meeting guidelines pre-pandemic. Patients with a home treadmill participated in twice the rate of calls compared to those without (RR = 2.22; 95%CI:1.35,3.64), but no other home environmental characteristics predicted participation rate. Pre-pandemic physical activity behavior appeared to predict pandemic physical activity and telephone-based physical activity support was effective for maintaining physical activity for some participants. Long term applications of this work will support continuity of clinic-community partnerships for health behavior change and provide a model for patient physical activity support by community health centers without on-site exercise facilities.

## Introduction

Although physical activity is important for everyone across the lifespan, physical activity is particularly essential to maintain physical and mental well-being during the COVID-19 pandemic. Physical activity enhances the circulation of immune cells, supports weight maintenance and can offset increased caloric consumption, reduces stress and anxiety, and improves sleep and quality of life [1–3]. Yet, emerging surveillance on physical activity during COVID-19 suggests that adults and youth worldwide participated in less physical activity and more sedentary time during the early-COVID-19 period [4, 5]. Health and safety guidelines for the COVID-19 pandemic reduced opportunities for physical activity in both indoor and outdoor public spaces. Exercise facilities closed to prevent exposure to the virus through close contact and touching surfaces of shared exercise equipment, many playgrounds and parks closed completely or severely limited occupancy due to social distancing guidelines, and team sports and activity classes for adults and youth were cancelled [6–8]. Although there was regional variation in duration and severity of COVID-19 shutdown measures, local shelter-in-place mandates undoubtedly limited physical activity opportunities outside of the home across the United States.

It is unclear how short-term changes in physical activity behavior and opportunities during 2020 may have affected long-term behavior and health outcomes. Accordingly, in a call to action for physical activity research to inform pandemic policies and practices, Sallis et al. [9] recommended research investigations focus on the impact of physical activity on COVID-19 symptoms and diagnosis, trends of human movement throughout the pandemic, locations for safe physical activity, and multilevel barriers and facilitators for maintaining physical activity throughout the pandemic, especially for those who were at an elevated risk for experiencing severe, adverse COVID-19 outcomes. Across all communities, culturally tailored physical activity promotion efforts can reduce health disparities among vulnerable populations [10]. Particularly during the COVID-19 pandemic, it was critical that physical activity promotion strategies facilitate equitable health opportunities, but was unclear if translating in-person physical activity promotion to distance-based efforts facilitated healthy behaviors. Thus, the purpose of this paper is to describe the reach and effectiveness of a telephone-based physical activity support and promotion strategy among racially/ethnically diverse patients at a Federally Qualified Health Center during the COVID-19 pandemic, and discuss lessons learned from adapting an in-person program to a telephone-based program that can be used to inform future behavior change strategies.

## Methods

### Study participants

Our study was conducted at a large, multi-site Federally Qualified Health Center (FQHC) in central Texas. The Heart of Texas Community Health Center has provided medical care in the community since 1970, operated as an FQHC since 1999, and does business as Waco Family Medicine. The Center provides medical, dental, and behavioral health care for almost 60 000 unique patients, or approximately 1 in every 5 county residents, each year. One-quarter (23%) of patients are non-Hispanic Black/African American, 48% are Hispanic/Latino, and 27% are non-Hispanic White. Approximately 1 in 6 patients do not have health insurance and are afforded care through a sliding scale discounted fee program that expands coverage to approximately one-quarter of self-pay patients.

### Study design

In 2017, Waco Family Medicine established the Wellness Center, an on-site exercise facility, to address limitations in accessible and affordable physical activity opportunities as part of a community-centered health home approach to wellness and health equity. The program has been described previously [11]. Briefly, patients receive “prescriptions” or referrals to the Wellness Center from their health care providers, initiate a visit at the Wellness Center, and meet one-on-one with personal fitness advisors. Fitness advisors have, at a minimum, a 4-year college degree in exercise physiology, CPR (cardiopulmonary resuscitation) certification, first aid certification, and continuing education training in motivational interviewing and the social determinants of health. The Wellness Center was designed as an in-person, short-term transitional facility to build confidence and teach strategies to support patient physical activity; then, patients would “graduate” or transition to other gym or exercise facilities in the community to maintain physical activity.

In March 2020, the Wellness Center closed in accordance with local COVID-19 health and safety guidelines. Fitness advisors pivoted from facilitating one-on-one, in-person advising at the Wellness Center to telephone-based support for physical activity. Fitness advisors (n = 4) contacted patients via telephone. Eligible patients had completed ≥1 in-person exercise advising visit at the Wellness Center in the month prior to the facility closure. During the initial telephone call, patients were asked to recall information about typical physical activity prior to the current coronavirus situation, describe resources at home that could facilitate physical activity while the Wellness Center was closed, and discuss exercise goals. Fitness advisors received data collection and motivational interviewing training and then used motivational interviewing techniques to help the patient develop Specific, Measurable, Attainable, Realistic, and Time-bound–or “SMART”–exercise goals [12]. Previous work has suggested that goal setting using the SMART framework can be used to modify health behaviors in many different settings, including increasing physical activity [13, 14]. As described in detail in the *Measures* section below, prompts and questions on a survey instrument guided the data collection.

Following the initial baseline telephone call, follow-up calls were made approximately every 2 weeks thereafter. Follow-up calls assessed physical activity behavior and helped patients set new or modified activity-related goals, once again with prompts and questions on a survey instrument for data collection. This report summarizes observational data from the telephone-based support for physical activity during the first 4 months of the telephone call program, from March 23, 2020 through July 23, 2020. The Baylor University Institutional Review Board defined Wellness Center surveys as program evaluation and therefore not meeting the definition of human subject research; however, verbal informed consent to participate in the program surveys was obtained from all participants.

### Measures

Patient sociodemographic and basic health characteristics were obtained from the electronic health record and included age (in years), sex (female or male), race/ethnicity (Hispanic or Latino, non-Hispanic white, non-Hispanic black, or non-Hispanic other), body mass index (BMI; in kilograms (kg) per meters (m)^2^), diabetes (yes/no), hypertension (yes/no), and any COVID-19 testing at Waco Family Medicine (yes/no).

Wellness Center utilization data were also obtained from electronic records. Previous utilization data included the date of exercise referral and a count of the total number of in-person visits to the Wellness Center. Date of initial intake visit less the date of Wellness Center closure was used to calculate duration of participation.

Baseline telephone survey data included self-reported physical activity and related health behavior theoretical constructs from the social cognitive theory, including self-efficacy and self-regulation strategies. At baseline, patients were asked to think about a typical week, prior to the COVID-19 pandemic. Patients were asked about any planned physical activity or exercise (yes/no) and days in a typical week they usually exercised at home (0–7 days). Physical activity was measured using a modified Baecke survey instrument that asked about type (free response), number of days, and duration of the two most frequently performed activities [15]. Open-ended responses to primary and secondary exercise activities were assigned a general metabolic equivalent of a task (MET) value using the Compendium of Physical Activities [16]. MET values were multiplied by frequency and duration to calculate average MET-minutes per week. Meeting physical activity guidelines was defined as ≥450 MET-minutes/week [17].

The Wellness Center program was designed to increase confidence for exercise and teach skills that support physically active lifestyles through one-on-one training sessions with fitness advisors [11]. Accordingly, social cognitive theory constructs, including self-efficacy and self-regulation strategies [18, 19], were used as a framework and assessed via survey. Self-efficacy for exercise was measured using a validated, six-item scale [20]. Patients were asked to rate on a scale of 1 to 5, with 1 indicating *not at all confident* and 5 indicating *extremely confident*, level of confidence that they could exercise when at home, when the weather is bad, when they do not have a fitness advisor, and make time for at least 30 minutes each day for physical activity (e.g., walking). Self-regulation strategies for exercise behavior change were measured using six questions [20]. Patients were asked to rate on a scale of 1 to 5, with 1 indicating *never* and 5 indicating *many times*, how often they kept track of physical activity, rewarded themselves for physical activity, told themselves that they could start again when physical activity plans got off track, have someone who encouraged them to do physical activity, set goals to do physical activity, and set aside time to exercise. Self-efficacy and self-regulation strategies were reported as mean scores on the six item scales, respectively.

Resources at home or “the place that you stay” were assessed with four yes/no questions about the presence of a safe space to walk outside, a treadmill or exercise bike, a kitchen chair, or free weights. Finally, patients were asked to identify goals for exercise while the Wellness Center was closed. Fitness advisors recorded the patient’s exact words. Then, fitness advisors used motivational interviewing strategies to help patients develop “SMART” goals [12], which were recorded on the survey instrument.

Follow-up telephone survey data also included self-reported physical activity data. First, patients were asked whether they did any planned physical activity or exercise (yes/no). Then patients were asked to report type (free response), number of days, and duration of the two most frequently performed activities, similar to the baseline survey. Patients were asked if they met their previous goal, and to report on their new goals. Once again, fitness advisors first recorded the patient’s exact words and then helped patients develop “SMART” goals.

### Statistical analysis

Statistical analyses were performed using SAS v9.4 (SAS Institute Inc, Cary, North Carolina, USA) and statistical significance was defined at the α = 0.05 level. First, descriptive statistics, including frequencies, means, and proportions, were generated for all sociodemographic and health variables in the total sample. Patient characteristics were compared across status, defined as *≥1 telephone survey*, *refused*, and *did not answer*, using chi-square tests for categorical variables and Kruskal-Wallis tests for continuous variables. Among patients with ≥1 telephone call, descriptive statistics were generated for patient data from survey responses and previous program utilization and then compared by meeting vs. not meeting physical activity guidelines status using chi-square tests for categorical variables and Kruskal-Wallis tests for continuous variables. To assess the relationship of patient characteristics and previous Wellness Center utilization with number of telephone calls, incident rate ratios were estimated using negative binomial regression (PROC GENMOD). Finally, we compared mean METs over time between those who were meeting vs. not meeting physical activity recommendations at baseline.

## Results

Between March 23, 2020 and July 23, 2020, 122 patients were contacted to participate in the telephone-based support program. Patients were, on average, 51.6 (±12.2) years of age, 86.1% female, 38.5% Hispanic or Latino, 16.4% non-Hispanic white, and 44.3% non-Hispanic black (see Table 1). More than half had hypertension (55.7%), 42.6% had diabetes, and most were obese. All patients who were contacted had utilized the in-person Wellness Center previously and participated in in-person exercise with fitness advisors prior to the pandemic. Number of days between original in-person Wellness Center program referral and Center closure due to COVID-19 was 195.8 (±197.1) days, meaning that patients had been participating in in-person exercise for approximately 6 months prior to the COVID-19-related closure. The mean number of previous in-person visits to the Wellness Center was 21.5 (±35.6) visits.

**Table 1:** Characteristics of patients with a Wellness Center phone call, by answer status, n = 122.

	Total	≥1 Phone Call	Refused	Did not answer	*P*
	n = 122	n = 72	n = 15	n = 35	
**Sociodemographics**					
Age, years (std)	51.6 (12.2)	51.3 (11.6)	55.1 (14.2)	50.8 (12.7)	0.54
Sex, %					
Male	13.9	12.5	6.7	20.0	0.44
Female	86.1	87.5	93.3	80.0	
Race/Ethnicity, %					
Hispanic or Latino	38.5	31.9	40.0	51.4	0.03
Non-Hispanic White	16.4	19.4	33.3	2.9	
Non-Hispanic Black	44.3	47.2	26.7	45.7	
Other	0.8	1.4	0.0	0.0	
Education, %					
Less than high school	13.5	15.3	8.3	11.1	0.84
High School / GED	36.0	39.0	33.3	27.8	
More than high school	50.6	45.8	58.3	61.1	
*Missing n = 33*					
Housing Insecurity, %	10.6	8.5	15.4	13.6	0.63
*Missing n = 28*					
Household Size, n (std)	3.3 (3.4)	2.8 (1.5)	4.0 (4.8)	4.2 (5.2)	0.80
Insurance					
Medicare	22.7	27.1	28.6	8.3	0.15
Medicaid or other public	32.0	28.8	28.6	41.7	
Private insurance	12.4	6.8	21.4	20.8	
None/uninsured	33.0	37.3	21.4	29.2	
**Health**					
BMI, kg/m^2^ (std)	36.4 (8.4)	37.1 (8.1)	32.7 (7.5)	36.4 (9.3)	0.12
Diabetes, %	42.6	44.4	33.3	42.9	0.80
Hypertension, %	55.7	55.6	60.0	54.3	0.93
**Previous program utilization**					
Days since program referral	195.8 (197.1)	166.2 (161.2)	220.6 (239.5)	246.0 (235.9)	0.63
Total in-person visits	21.5 (35.6)	17.8 (24.7)	42.3 (78.6)	20.2 (21.6)	0.66
**COVID data**					
Ever tested, %	13.9	15.3	6.7	14.3	0.86

Of the 122 patients with contact attempts, 72 patients (72/122; 59%) participated in ≥1 phone call with a fitness advisor, 15 patients declined participation, and 35 could not be reached via telephone. Non-Hispanic white patients were more likely to refuse participation, non-Hispanic black patients were less likely to refuse participation (p = 0.03). No other differences across participation status reached statistical significance at the α = 0.05 level.

Among the 72 patient participants, the mean number of days between the local shelter-in-place order and the initial phone call was 18.7 (±11.7) days. Almost three-fourths (70.8%) planned physical activity during a typical week prior to the COVID-19 situation and exercised at home, on average, 2.9 days ± 2.6, during a typical week. Approximately one-third of patients met physical activity guidelines. Most (91.6%) reported a safe space at home to walk outside, 16.9% reported a treadmill, 85.7% reported ownership of a kitchen chair, and 46.5% reported ownership of free weights.

Among patients who were meeting physical activity guidelines at baseline (n = 25), all planned physical activity during a typical week. Those meeting physical activity guidelines reported significantly more days of exercise at home prior to COVID-19 shutdown (5.0 days vs. 1.7 days; p<0.001) and significantly higher self-efficacy (4.0 vs. 3.2; p = 0.01) than patients not meeting physical activity guidelines (Table 2).

**Table 2:** Characteristics of patients with ≥1 Wellness Center phone call, by physical activity status at baseline, n = 72.

	Total	Meeting PA Guidelines	Not meeting PA Guidelines	*P*
	n = 72	n = 25	n = 47	
**Physical activity prior to pandemic**				
Any planned activity during typical week, %	70.8	100.0	55.3	<0.001
Exercise at home during typical week, days (std)	2.9 (2.6)	5.0 (2.2)	1.7 (2.0)	<0.001
METs, mean (std)	486.0 (637.9)	1133.1 (697.4)	141.7 (160.1)	<0.001
Meeting PA guidelines, %	34.7	---	---	
Self-efficacy, mean (std)	3.5 (1.0)	4.0 (0.7)	3.2 (1.1)	0.01
Self-regulation, mean (std)	3.1 (0.9)	3.2 (0.9)	3.0 (0.8)	0.27
**Home resources**				
Safe space to walk outside, %	91.6	96.0	89.1	0.41
Treadmill or exercise bike, %	16.9	24.0	13.0	0.32
Kitchen chair, %	85.7	92.0	82.2	0.26
Free weights, %	46.5	56.0	41.3	0.32
**Program engagement**				
Days between shelter in place and first phone call, mean (std)	18.2 (11.1)	17.7 (11.4)	18.5 (11.4)	0.47
Total number of calls, mean (std)	3.8 (2.1)	4.2 (2.6)	3.5 (1.8)	0.23
Proportion of visits meeting PA goals	45.3	57.0	39.7	0.11
Days between phone calls, mean (std)	24.4 (14.5)	22.6 (10.2)	25.3 (16.1)	0.50

Notes: Meeting physical activity (PA) guidelines defined as ≥450 MET-minutes during a typical week prior to the pandemic shelter-in-place; METs estimated from self-reported survey data.

**Fig 1:**
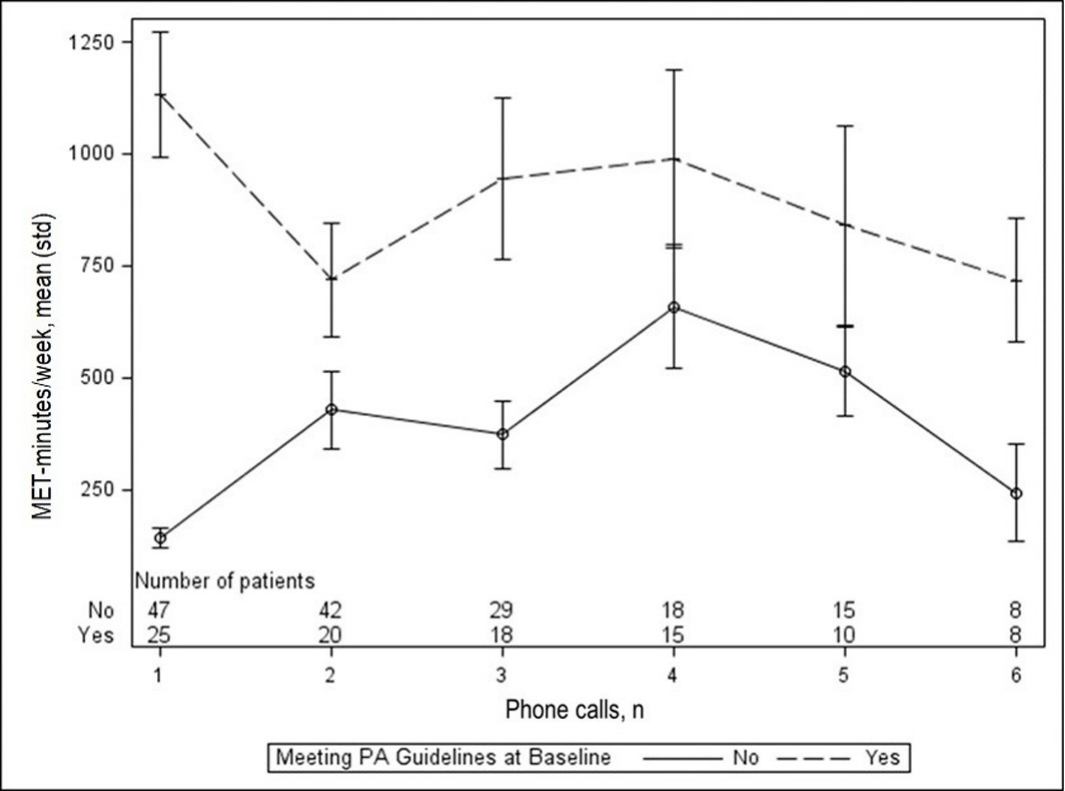
MET-minutes per week, by phone call and meeting vs. not meeting physical activity (PA) guidelines at baseline, n = 72.

During the first 4 months of the telephone call program, fitness advisors completed 270 phone calls. The number of calls per patient participant ranged from 1 to 9 (mean = 3.8 ± 2.1; median = 3.0). Patients who had a treadmill at home participated in approximately twice the rate of telephone calls compared to those who did not (RR = 2.22; 95% CI: 1.35, 3.64; p = 0.002), but no other physical activity behavior prior to the pandemic, home resources, or previous program engagement variable was a statistically significant predictor of telephone call participation rate. Participants reported meeting physical activity goals during almost half (45.3%) of their telephone calls. Patient participants who were meeting physical activity guidelines before the COVID-19 shutdown of the Wellness Center appeared to maintain activity at or above the recommended level and patient participants who were not meeting physical activity guidelines appeared to experience an increase in activity through 4 telephone calls followed by a decline (Fig 1).

## Conclusions

During the COVID-19 pandemic, an in-person exercise prescription program was adapted to a telephone-based support for physical activity. At a Federally Qualified Health Center, 59% patients who were contacted about the telephone-based physical activity goal setting program engaged in ≥1 telephone call, and of those who engaged in ≥1 telephone call, 65% completed ≥3 phone calls. The RE-AIM policy planning and evaluation framework considers five dimensions for evaluating public health impact: reach, efficacy/effectiveness, adoption, implementation, and maintenance [21, 22]. In our study, we specifically considered reach, defined as the absolute number, percentage, or representativeness of patients affected by the telephone-based program; and effectiveness, defined as the change in proximal outcomes associated with telephone-based program. RE-AIM can be useful for comparing health promotion programs [22]. As such, comparisons of an in-person exercise prescription program and a telephone-based prescription program can be useful for future resource planning. In our organization, we observed a slightly higher reach with the telephone program (59% vs. 52% [11]), but the in-person program had higher engagement and number of contacts with fitness advisors, as previously published [11].

We expected prior in-person program engagement to be a bellwether for telephone program engagement; however, previous program engagement and number of days since initial Wellness Center referral were not associated with telephone program engagement. From March 23 through July 23, 2020, it appears that patients who were meeting physical activity guidelines prior to the COVID-19-related shutdown maintained recommended levels of physical activity, and patients who were not meeting physical activity guidelines experienced a temporary increase in activity.

Multiple studies have demonstrated that past physical activity behavior predicts future physical activity behavior [23, 24]. Past experiences have direct and indirect effects on self-efficacy, which plays a critical role in the initiation and adoption of health behaviors [25]. In our study, patients who were meeting physical activity guidelines prior to the COVID-19 pandemic reported higher self-efficacy than patients who were not meeting physical activity guidelines. Adults who were meeting physical activity guidelines planned more activity during a typical week and reported more at-home exercise days pre-pandemic, which likely set them up for physical activity success during COVID-19 stay-at-home orders. Nevertheless, we observed a decline in activity from the first to the second telephone calls for adults who were meeting physical activity guidelines at baseline. This observation is consistent with smartphone tracking data from the United Kingdom, which showed that there was a dramatic decline in physical activity among those who were fairly active at baseline [26].

Motivational interviewing and goal setting were important components of this telephone support intervention. When patients were asked initially about their goals for exercise, many reported their goal was, e.g., “to be more active.” Fitness advisors worked with patient participants to develop Specific, Measurable, Attainable, Realistic, and Time-bound goals, or “SMART” goals, e.g., “walk for 30 minutes, five times per week.” These individualized goals were developed with patients based on their home resources and in-person exercise experience at the Wellness Center prior to the pandemic. Goal setting is an important component of behavior change, as described by the Social Cognitive Theory, because goals mediate the relationship between self-efficacy and physical activity [19, 27]. During this four-month observation period, patients reported meeting personal activity goals during approximately half of all telephone visits. Perhaps unsurprisingly, adults who were meeting physical activity guidelines at baseline had a slightly higher proportion of calls for which they reported meeting physical activity goals during the telephone program than adults who were not meeting physical activity guidelines at baseline (57.0% vs. 39.7%; p = 0.11). At baseline, patients who were meeting physical activity guidelines reported significantly higher self-efficacy than patients who were not meeting physical activity guidelines. Patients who were meeting guidelines also maintained higher levels of activity and had proportionally higher study retention throughout the four-month observation period, which may be explained by higher baseline self-efficacy.

Our telephone support program is an example of a distance-based approach to physical activity intervention. Distance-based approaches are “characterized by limited face-to-face contact and/or supervision and are typically delivered outside of the clinical setting…[and] may involve intervention modalities including print materials, telephone counseling, text messaging, mobile apps, and web-based platforms” [28]. A number of studies have reported the utility of distance-based interventions. For example, among adolescents, mobile phone-based interventions increased short-term physical activity [29], and among older adults, interventions utilizing text messaging and mobile phones apps increased physical activity and decreased BMI among overweight individuals [30]. Distance-based physical activity interventions may be useful among race/ethnic minority populations in particular. Among Hispanic/Latino adults with type 2 diabetes, text messaging, phone calls, and recorded voice messages to encourage physical activity resulted in increased steps per day and an increased perception of social support [31, 32]. Among African American women, feedback on the feasibility of a mobile phone app-based intervention to increase physical activity suggested that women used their phones daily and viewed them as a tool of empowerment and safety which would likely increase their physical activity over time [33]. In another study of African American women, weekly phone calls with health coaching significantly increased physical activity and decreased BMI over 24 months [34]. However, regardless of the technology modality, individualized feedback is necessary for participant engagement and sustainable behavior change [35].

In our study, approximately 80% of patients who participated in ≥1 telephone call identified as Hispanic/Latino or non-Hispanic Black. This race/ethnic distribution is in keeping with the overall patient population of the clinic system, and over 90% of patients live at or below 200% of the federal poverty guidelines. In this study, we surveyed patients about home resources and their environments because we previously described the community served by our clinic as an *exercise desert*, a built environment with limited walkability, green space, and affordable fitness facilities [11, 36–39]. Limited access to safe outdoor spaces, coupled with greater life demands, negatively influence physical activity in low-income communities [38–40], and low-income adults and those from race/ethnic minority groups have been disproportionately affected by the health and economic effects of COVID-19. Future work should explore theory- and distance-based modalities, including text messaging and apps, for physical activity health coaching to address health disparities.

Our study is subject to several limitations. First, we only used a telephone-based physical activity strategy. Although internet and mobile phone-based interventions that allow for audio-visual connections can improve physical activity, socioeconomic disparities in internet access limited the accessibility of interventions that required the internet [35]. Also, approximately 28% of patients who were eligible to participate could not be reached via telephone, despite a clinic protocol to confirm a working telephone number at every clinical encounter. We also observed attrition during the course of the observation period, and we do not have information about the reason(s) for drop out. In our community, the local stay-at-home mandate began on March 23, 2020 and was relaxed on May 1, 2020, for 39 total days. On average, the first telephone call occurred 18.2 ±11.1 days after the shelter-in-place mandate, the second telephone call occurred 34.1±17.8 days after the mandate, and the third telephone call occurred 59.0 ±25.8 days after the mandate. Attrition and/or change in behavior that occurred between the second and third calls may have been due to changes in local policies, although we did not collect information about personal behavior or employment. We also did not collect information about mental health or depressive symptoms, the need for which was unforeseen in March 2020 when the short survey was developed. Although we have information about COVID-19 testing during our four-month observation period, it is limited to data from electronic health records and does not include testing from alternative-site community-wide surge testing. Approximately 14% of the 122 participants received a COVID-19 test at the clinic during the four-month observation period, but we are aware of fewer than 5 positive cases among our participants during this time (results are suppressed for anonymity due to the small sample size), which indicates that our community was experiencing low community spread during the observation period. Geographic and calendar variation in COVID-19 epidemiology and policies may limit the generalizability of our work. Finally, we do not have direct or device-based measurement of physical activity. Our work relies on self-reports of physical activity, and while common in epidemiologic studies, is subject to known error. Furthermore, MET estimates may not reflect individual energy expenditure [41, 42].

Nevertheless, we believe this work has three primary applications. Internally and immediately, telephone-based support for physical activity facilitated support of health behaviors during COVID-19, which achieved continuity of fitness advisor-patient interactions. Second, the Wellness Center was originally envisioned as a temporary plan for patients to build exercise-related skills and confidence before transitioning to other community exercise facilities. The lessons learned here–for example, the availability and relative importance of home resources among our patient population–will allow us to develop best practices for telephone-based transitional support after Wellness Center “graduation” when patients move to other exercise facilities in our community in the future. We also plan to explore the utility of video-based support and messaging through the electronic health system (e.g., “MyChart”) for transitional support. Third, our Community Health Center has a unique on-site exercise facility to support equitable health opportunities through a community-centered health home approach to population health. Even beyond COVID-19 and exercise facility closure, other health centers may wish to implement telephone-based support for physical activity in the absence of an on-site exercise facility. Clinic-community partnerships for physical activity support can build on these findings and our prior work [11] to support health equity.

During the COVID-19 pandemic, low-income adults were disproportionately affected by the virus and related economic sequelae, including increased exposure to the virus due to “essential worker” working conditions, increased caregiver burden and decreased discretionary time, and increased burden of comorbidities [43, 44]. Although not a panacea, physical activity is an important component of maintaining physical and mental health, particularly for a low-income patient population disproportionately affected by the health and economic sequelae of COVID-19. More work is needed to identify long-term pattern changes in physical activity worldwide. Telephone-based support for physical activity at a Federally Qualified Health Center may lessen structural barriers to physical activity and provide support for healthy behaviors both during and after the pandemic.

## Supporting information

S1 File (PDF)
