# Supplementary material for: Telephone-based support for physical activity: Results and lessons learned during the COVID-19 pandemic
Source: PLoS One. 2022 May 18;17(5):e0268429. doi: 10.1371/journal.pone.0268429 (PMC9116679; doi:10.1371/journal.pone.0268429)
Supplement: S1 File — (PDF) [file pone.0268429.s001.pdf]

NAME: __ *Write exact response*__________DOB:__*MM/DD/YYYY*_________TODAY'S DATE:___*MM/DD/YYYY*__________

1. Think about a typical week, <u>prior to</u> the current coronavirus situation.

In a typical week, do you do planned physical activity or exercise? **1** Yes **2** No [Skip to 3]

Which exercise do you do most frequently? _*Write exact response*__________________________________

How many days in a typical week do you do this activity?
0 None **1** 1 day **2** 2 days **3** 3 days **4** 4 days **5** 5 days **6** 6 days **7** 7 days

How much time in a typical day do you do this activity? __ *Write exact response* ___ minutes

When you do this activity, do your heart rate and breathing increase?
0 No **1** Yes, a small increase **2** Yes, a moderate increase **3** Yes, a large increase

What is the second most frequent exercise you do? __ *Write exact response* ____________________

How many days in a typical week do you do this activity?
0 None **1** 1 day **2** 2 days **3** 3 days **4** 4 days **5** 5 days **6** 6 days **7** 7 days

How much time in a typical day do you do this activity? _ *Write exact response* __ minutes

When you do this activity, do your heart rate and breathing increase?
0 No **1** Yes, a small increase **2** Yes, a moderate increase **3** Yes, a large increase

2. Think about a typical week, <u>prior to</u> the current coronavirus situation.

How many days in a typical week do you usually exercise at home?

0 None **1** 1 day **2** 2 days **3** 3 days **4** 4 days **5** 5 days **6** 6 days **7** 7 days

3. Think about a typical week, <u>prior to</u> the current coronavirus situation. On a scale of 1 to 5, with 1 being never and 5 being many times, please rate how often you…

	Never	Almost Never	Some-times	Often	Many Times
You keep track of how much physical activity you do.	1	2	3	4	5
You reward yourself for being physically active.	1	2	3	4	5
You tell yourself that you can start again when your physical activity plans get off track.	1	2	3	4	5
You have someone who encourages you to do physical activity.	1	2	3	4	5
You set goals to do physical activity.	1	2	3	4	5
You set aside time to exercise.	1	2	3	4	5

4. Think about a typical week, prior to the current coronavirus situation. On a scale of 1 to 5, with 1 being not at all confident and 5 being extremely confident, please rate how confident you are that you can exercise…

	Not at all Confident	Somewhat Confident	Moderately confident	Very Confident	Extremely confident
…When you are at home.	**1**	**2**	**3**	**4**	**5**
…When the weather is bad.	**1**	**2**	**3**	**4**	**5**
…When you do not have a fitness advisor with you.	**1**	**2**	**3**	**4**	**5**
Make time for at least 30 minutes each day of physical activity, like walking.	**1**	**2**	**3**	**4**	**5**

5. Think about the place that you stay. What things do you have at home that could help you be active?

A safe space to walk outside? **1** Yes **2** No
A treadmill or exercise bike at home? **1** Yes **2** No
A kitchen chair? **1** Yes **2** No
Free weights? **1** Yes **2** No
Other: _ *Write exact response* ________________________

6. The Wellness Center is closed during the current coronavirus situation. What are your goals for exercise while the Wellness Center is closed? (*Please help the patient articulate their goal as a SMART goal*.)

Patient's exact words:	SMART goal:
Write exact response	*Write exact response*

7. EXERCISE PRESCRIPTION. Next week, you should do this exercise:

Write exact response

8. FITNESS ADVISOR NOTES:

Write exact response
